# Reshaping the cardiovascular continuum in the management of arterial and venous cardiovascular disease: a narrative review

**DOI:** 10.57264/cer-2025-0162

**Published:** 2026-01-23

**Authors:** Khadija Hafidh, Melina Vega de Ceniga, Leonardo De Luca, Claudio Borghi

**Affiliations:** 1 Dubai Academic Health Corporation, Dubai, UAE; 2 University Hospital of Galdakao-Usansolo, Bizkaia, Spain; 3 Fondazione IRCCS Policlinico San Matteo, Pavia, Italy; 4 University of Bologna, Bologna, Italy

**Keywords:** adherence, cardiometabolic disease, chronic venous disease, coronary artery disease, integrated care, multidisciplinary management, peripheral arterial disease, therapeutic inertia

## Abstract

The prevalence of cardiovascular disease (CVaD) is expected to double in the next 25 years, fueled by increasing prevalence of diabetes mellitus, obesity and hypertension. Cardiovascular–kidney–metabolic syndrome is a clinical entity requiring a holistic approach to prevention and management. Another aspect of this syndrome is chronic venous disease (CVeD), which is common in patients with CVaD. This review describes presentations at a symposium by the European Association of Preventive Cardiology (Milan, Italy; April 2025), discussing the interconnectedness of conditions on the CVaD continuum and their relationship with CVeD. Venous and arterial disease share common risk factors and pathogenic pathways, including endothelial dysfunction, increased vascular permeability, oxidative stress and inflammation. Many cardiometabolic and vascular conditions remain underdiagnosed and untreated, and the patients’ level of risk is often underestimated. Examination of the legs is important to identify peripheral arterial disease and/or CVeD. The mainstays of treatment for CVeD are exercise, compression therapy, venoactive drugs and surgery. Failure to achieve and maintain treatment goals is usually the result of therapeutic inertia or poor medication adherence. A coherent approach is needed to identify and manage shared risk factors and comorbidities. Effective disease management and risk reduction require integrated care using multidisciplinary teams; evidence-based treatments, usually with combination therapy; and use of tools to maximize adherence, including digital tools and single-pill combinations to simplify treatment regimens in patients with multiple risk factors or comorbidities.

## Plain language summary: a narrative review of the management of arterial & venous cardiovascular disease

**What is this article about?:**

This article describes the common features of cardiovascular disease (CVaD) and chronic venous disease (CVeD) and encourages a holistic approach to their management. This includes actively looking for CVeD in patients with CVaD (and *vice versa*) and prescribing effective treatments for both conditions. The article also raises awareness of the effect on health outcomes of adherence (patients taking treatment regularly as prescribed) and therapeutic inertia (doctors failing to appropriately escalate treatment to meet patient needs).

**Why is this important?:**

Risk factors for CVaD, such as obesity, diabetes and hypertension (high blood pressure), affect the structure and function of blood vessels (arteries and veins). The resulting damage to arteries has a negative effect on the heart and kidneys, and eventually causes significant health events, such as stroke, heart attack or kidney failure. Damage to veins causes CVeD, which is associated with varicose veins, leg pain, skin problems and leg ulcers. Although CVaD and CVeD are closely related, many doctors do not see them that way.

**What methodology/protocol is described?:**

A symposium on CVaD and CVeD was held at the European Association of Preventive Cardiology annual congress in Milan, Italy on 4 April 2025. This article highlights the presentations at the symposium.

**What were the results?:**

In both CVaD and CVeD, therapeutic inertia can be overcome by actively diagnosing risk factors, managing risk factors and symptoms with evidence-based therapies, monitoring the effect of treatment on the patient, and changing the medication if treatment goals are not being met. Adherence can be maximized by choosing medication that is easy for patients to take (e.g., once daily medications, single-pill combinations), using digital tools to deliver reminders and feedback, and investigating and removing (where possible) other barriers that affect a patient’s medication-taking behavior.

## Shareable abstract

This article describes presentations at a symposium held at the European Association of Preventive Cardiology annual congress on reducing the risk and optimizing the management of cardiovascular disease, which often occurs with chronic venous disease, in patients with multiple risk factors or comorbidities.

Cardiovascular disease (CVaD) is a significant global health issue, responsible for 30% of global deaths and affecting ∼598 million people in 2025; this prevalence is expected to double in the next 25 years, with increases in every region of the world [1].

The pathogenic process of CVaD is a continuum in which risk factors develop and accumulate, causing vascular and end-organ damage that becomes symptomatic and irreversible [2]. The global increase in CVaD incidence is being fueled by the twin epidemics of ‘diabesity’ (diabetes and obesity) and hypertension [3], key modifiable risk factors for which there are effective treatments.

There are multiple steps along the pathogenic continuum in which patient outcomes can be modified, by identifying risk factors, prescribing appropriate treatments and following up patients regularly to evaluate their ongoing adherence, health and well-being. In addition to the ‘classical’ CVaD risk factors of age, sex, smoking, hyperlipidemia, hypertension, obesity, renal dysfunction and diabetes, there is growing recognition that an unhealthy diet, a sedentary lifestyle, ethnicity and socioeconomic status also contribute to CVaD risk [4]. Risk factors are often shared between cardiovascular, renal and metabolic diseases. The presence of additional conditions, such as obesity in a patient with CVaD or renal disease in a patient with diabetes, accelerates and amplifies the progression of all three [5]. This has led to increasing recognition of cardiovascular–kidney–metabolic syndrome as a clinical entity requiring a holistic approach to patient management [5,6]. There is also growing evidence that disease in an arterial vascular bed is commonly associated with chronic venous disease (CVeD) [7]. Yet, unfortunately, data show that many of the opportunities for effective intervention are being missed [8].

A symposium was held at the meeting of the European Association of Preventive Cardiology in Milan, Italy on 4 April 2025, discussing the interconnectedness of conditions on the CVaD continuum and their relationship with CVeD. The current narrative review describes the content of that symposium, focusing on ways in which the clinical paradigm for CVaD needs to be re-examined and how healthcare professionals (HCPs) can optimize patient management to address the global burden of CVaD and CVeD.

## Materials & methods

This review is based on the presentations at the symposium. The speakers at the symposium (and authors of this review) conducted literature searches of PubMed to identify articles relevant to their presentations. Additional ad hoc searches of PubMed were undertaken during the development of the review to support specific statements.

### Peripheral & central vascular disease

Peripheral arterial disease (PAD) is a well-established marker of CVaD and a predictor of cardiovascular death, principally because disease in one vascular bed (such as the lower extremity) is often a marker of disease in another (such as the coronary or carotid arteries) [9]. Among patients with intermittent claudication, within 5 years of diagnosis 1.1% will progress to chronic limb-threatening ischemia, but only about 0.2% will require major limb amputation (due to improvements in limb salvage) [10]. However, the 5-year mortality rate is 19–26.7% [10–12], with CVaD the leading cause of death in patients with PAD [10,12]. Unlike the reduction in coronary artery disease (CAD) mortality rates seen in the last 20 years, the PAD mortality rate has not significantly changed, partially because of late diagnosis with higher atheroma burden, but also because of underdiagnosis and undertreatment [9].

#### Peripheral venous disease & CVaD

Peripheral vascular disease affects not only arterial circulation but venous circulation too; indeed, CVeD is more prevalent than CVaD. CVeD is classified using the clinical, etiological, anatomical, pathophysiological (CEAP) system with the clinical severity graded from C0 (no visible or palpable signs of venous disease) to C6 (active venous ulcer) [13]. Prevalence of some degree of CVeD (CEAP grade C1–C6) was 63.7% when estimated using data from the Vein Consult program involving 23 countries, and the prevalence of chronic venous insufficiency (CVI; CEAP grade C3–C6) was 26.0% [14]. Importantly, preclinical venous disease can be present from a young age: the Bochum study detected reflux in the great saphenous vein in 2.4% of 11- to 12-year-olds and 10.4% of 14- to 16-year-olds [15].

Venous disease tends to progress quickly. About 30% of the young people in the Bochum study with reflux went on to develop truncal varicose veins within 4 years [15]. In the Edinburgh Vein study, the annual rate of progression among patients with CVI or varicose veins was 4.3% [16]. A different study reported 22% of patients with untreated varicose veins (CEAP grade C2) progressing to a venous ulcer (CEAP grade C6) within 6 years [17].

**Figure 1.**
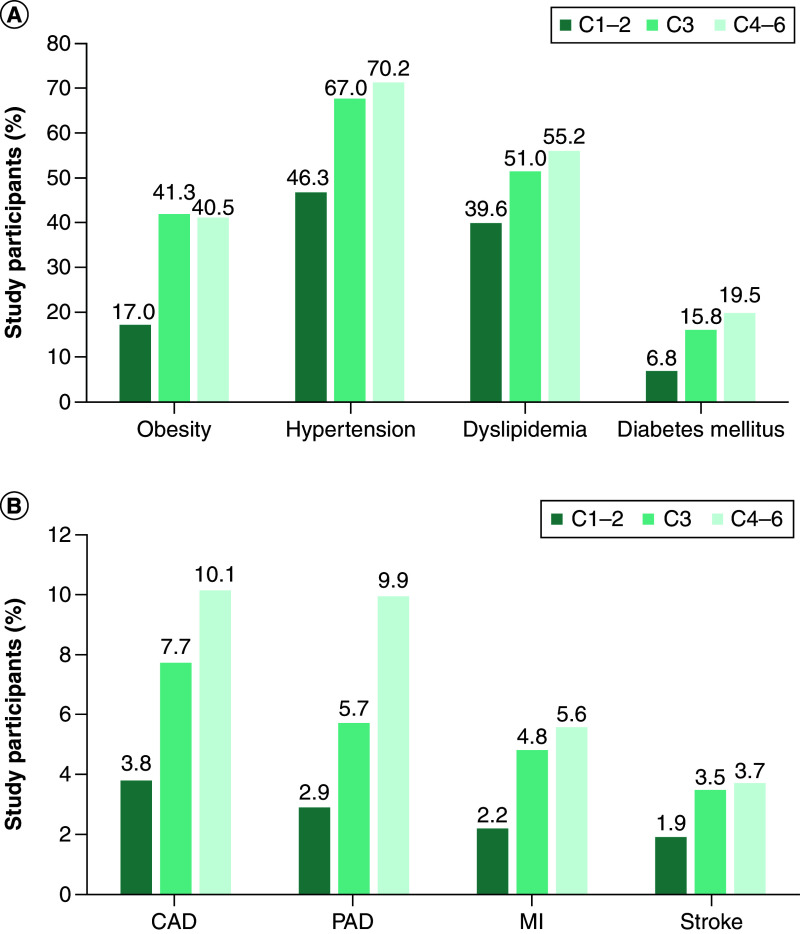
The prevalence of cardiovascular risk factors and cardiovascular disease increases with worsening clinical, etiological, anatomical and pathophysiologica grade in patients with chronic venous disease. Data are from the Gutenberg Health Study. **(A)** Cardiovascular risk factor prevalence. **(B)** Cardiovascular disease prevalence [18]. C1: Telangiectasias or reticular veins; C2: Varicose veins; C3: Oedema; C4: Skin or subcutaneous changes; C5: Healed venous ulcer; C6: Active venous ulcer. CAD: Coronary artery disease; CEAP: clinical, etiological, anatomical, pathophysiological; MI: Myocardial infarction; PAD: Peripheral arterial disease. Obesity was defined as body mass index >30 kg/m^2^.

There is accumulating evidence for an association between CVeD and CVaD [18–21]. For example, the Gutenberg Health Study examined the relationship between CVI and CVaD in 12,423 people aged 40–80 years [18]. The majority of patients (91.5%) had some type of CVeD (CEAP grade C1 or higher) and 43.2% had CVI (CEAP grade C3–C6). The prevalence of both cardiovascular risk factors (Figure 1A) and CVaD (Figure 1B) increased with worsening CEAP grade. Patients with more severe CVI (CEAP grades C4–C6) comprised 11.7% of the population. At least 70% of this latter group had hypertension, 40% were obese, almost 20% had diabetes, 10% had CAD and 10% had PAD [18]. Indeed, CVI (CEAP grades C3–C6) was a significant independent predictor of CVaD (p = 0.002), even after adjustment for age, sex and traditional risk factors (hypertension, diabetes, dyslipidemia, family history of CVaD, obesity and smoking) [18]. Patients with more severe CVI had a 10-year CVaD event risk of 23.3%. CVI was also a significant predictor of all-cause death (p < 0.0001), even after adjustment for age, sex and traditional risk factors, and medication use, in the overall population and in the subgroup of patients without prevalent CVaD at baseline (p = 0.006) [18].

### Paradigm shift

**Figure 2.**
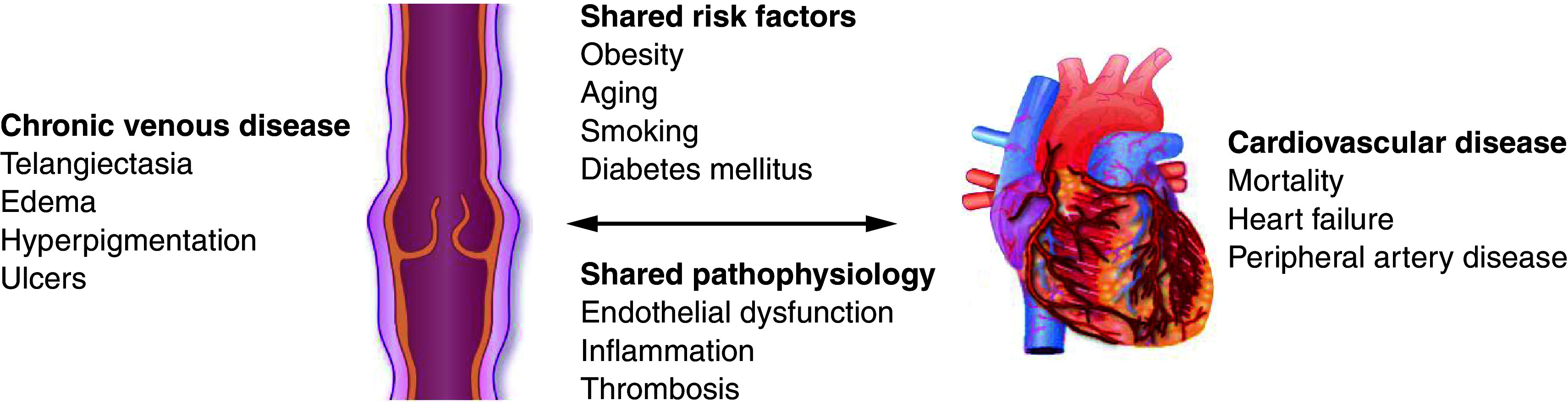
Shared risk factors and pathogenic processes in arterial and venous vascular disease. Reproduced by permission of Oxford University Press on behalf of the European Society of Cardiology from Hamburg NM [7].

**Figure 3.**
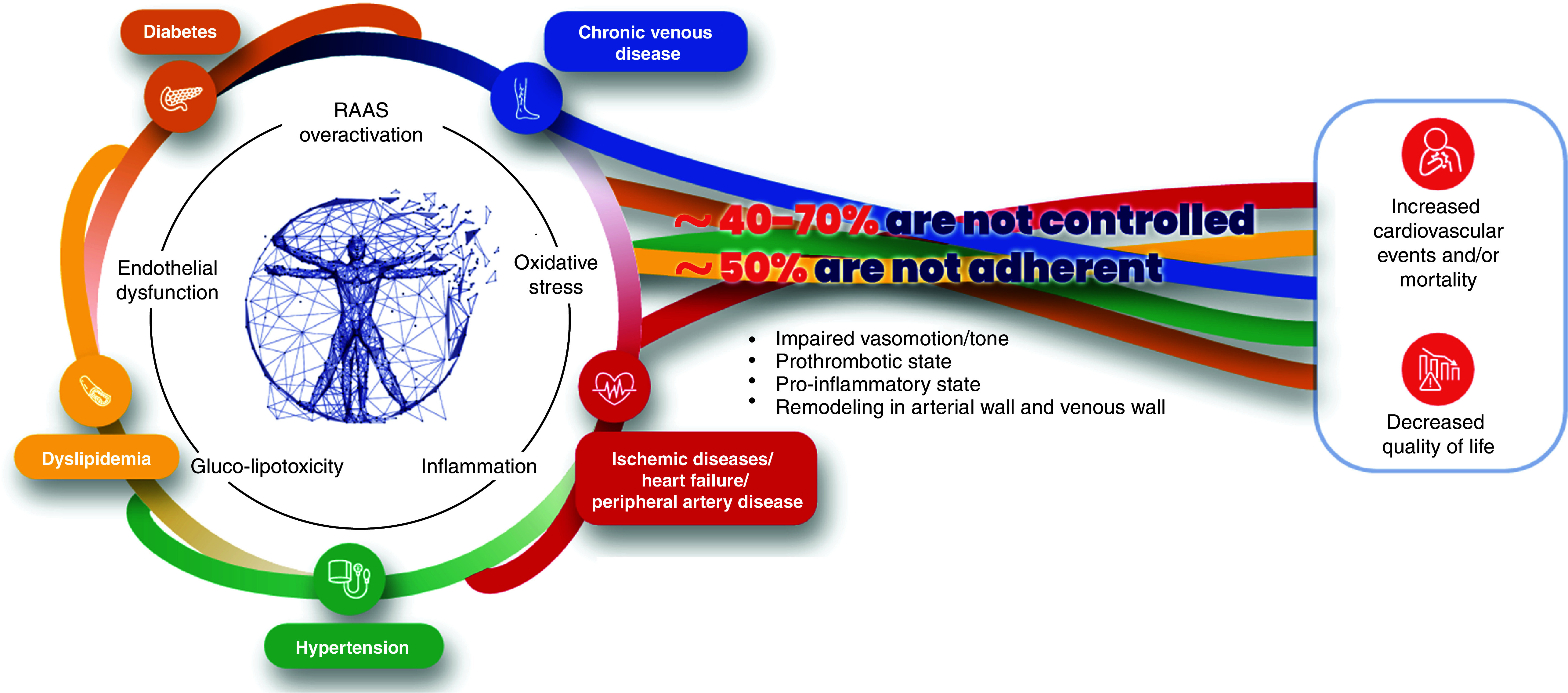
The interconnectedness of arterial and venous disease, and cardiometabolic risk factors, warrants a paradigm shift in which all of these are seen as inter-related elements of the same pathogenic process requiring effective treatment to improve outcomes. Data used to create this figure are from several sources [7,22,46–50]. © 2025 Les Laboratoires Servier – all rights reserved. RAAS: Renin–angiotensin–aldosterone system.

Venous and arterial disease not only share common risk factors, but they also share common pathogenic pathways (Figure 2), including endothelial dysfunction, increased vascular permeability, oxidative stress, renin–angiotensin–aldosterone system (RAAS) activation and inflammation [7,22,23] (Figure 3). The interconnectedness of these conditions highlights the need for a paradigm shift in the management of CVaD, in which a more coherent approach is taken to the identification and management of shared risk factors and comorbidities. Yet, many cardiometabolic and vascular conditions remain underdiagnosed [24–26] and therefore untreated. Among those who are treated, achievement of guideline-recommended goals is consistently low for most risk factors (in anywhere from 40 to 70% of patients, Figure 3), including low-density lipoprotein (LDL) cholesterol [27–31], blood pressure (BP) [29,32,33] and glycated hemoglobin (HbA1c) [34,35]. Failure to achieve and maintain risk factor targets is usually the result of therapeutic inertia or poor medication adherence [36], which may be prevalent in up to 50% of patients (Figure 3). Not meeting such targets increases the risk of morbidity and mortality [37–45].

#### Holistic treatment of CVeD

The key guideline-recommended therapies for CVeD are exercise and lifestyle changes (class IIa recommendation; level of evidence B), compression therapy (class IIa recommendation; level of evidence B), venoactive drugs (class IIa recommendation; level of evidence A) or surgery (class I recommendation; level of evidence B or C) [51]. All of these treatments are aimed at reducing venous hypertension, and they reduce inflammation either directly (as exercise and many venoactive drugs do) or indirectly as a secondary effect of reducing venous pressure [52].

Venoactive drugs target cellular pathophysiological cascades, similarly to medications for arterial hypertension, hypercholesterolemia and diabetes mellitus, and as such, are a highly effective class of therapy for CVeD. Venoactive agents improve the symptoms of venous disease (Table 1) through various mechanisms of action (Table 2) [51–53], mainly improving vascular tone, reducing venous capillary leakage and having an antioxidant effect [52]. More data support the use of micronized purified flavonoid fraction versus other venoactive agents [46]. The question remains as to whether these agents could have similar beneficial effects on arterial endothelium and as such, help reduce cardiovascular risk in patients with venous disease. Studies are urgently required to address this question.

**Table 1.** The signs and symptoms of chronic venous disease positively impacted by different venoactive drugs.

MPFF	Anthocyanins (red vine leaf extracts)	Rutosides	Ruscus extracts	Horse chestnut extract	Calcium dobesilate
Pain, heaviness, feeling of swelling, discomfort, cramps, paresthesia, burning, redness, skin changes/trophic disorders, oedema, venous ulcer	Pain, oedema	Pain, heaviness, cramps, swelling, paresthesia, pruritus, oedema	Pain, heaviness, feeling of swelling, fatigue, cramps, paresthesia, pruritus, oedema	Pain, pruritus, oedema	Pain, heaviness, discomfort, fatigue, cramps, swelling, restless legs, paresthesia, pruritus, oedema

Adapted from De Maeseneer MG *et al.* [51], licensed under CC BY 4.0 (https://creativecommons.org/licenses/by/4.0/). Changes include minor changes to column titles, selection of drugs represented in table, and data are shown using text rather than tick-mark symbols. Additional effects are shown for some drugs based on information from other sources [52,53].

MPFF: Micronized purified flavonoid fraction.

**Table 2.** The mechanisms of action of the key venoactive drugs.

Venoactive drugs	Evidence exists for effects on					
	Venous tone	Venous wall and valve	Capillary leakage	Lymphatic drainage	Hemorheological disorders	Free radical scavenging
Flavonoids (gamma-benzopyrones)						
MPFF	✓	✓	✓	✓	✓	✓
Non-micronized or synthetic diosmins	No data	No data	No data	No data	No data	No data
Rutin and rutosides	✓		✓	✓	✓	✓
Anthocyanins (*Vitis vinifera*)						✓
Proanthocyanidins (*Vitis vinifera*)			✓			✓
Alpha-benzopyrones						
Coumarin			✓	✓		
Saponins						
Horse chestnut seed extract (aescin)	✓		✓			
Ruscus extract	✓		✓	✓	✓	
Other plant extracts						
Gingko extracts	No data	No data	No data	No data	No data	No data
Synthetic products						
Calcium dobesilate	✓		✓	✓	✓	✓
Benzarone	No data	No data	No data	No data	No data	No data
Naftazone	No data	No data	No data	No data	No data	No data

MPFF: Micronized purified flavonoid fraction.

Reprinted by permission of Edizioni Minerva Medica [52].

### The challenge of multimorbidity

**Figure 4.**
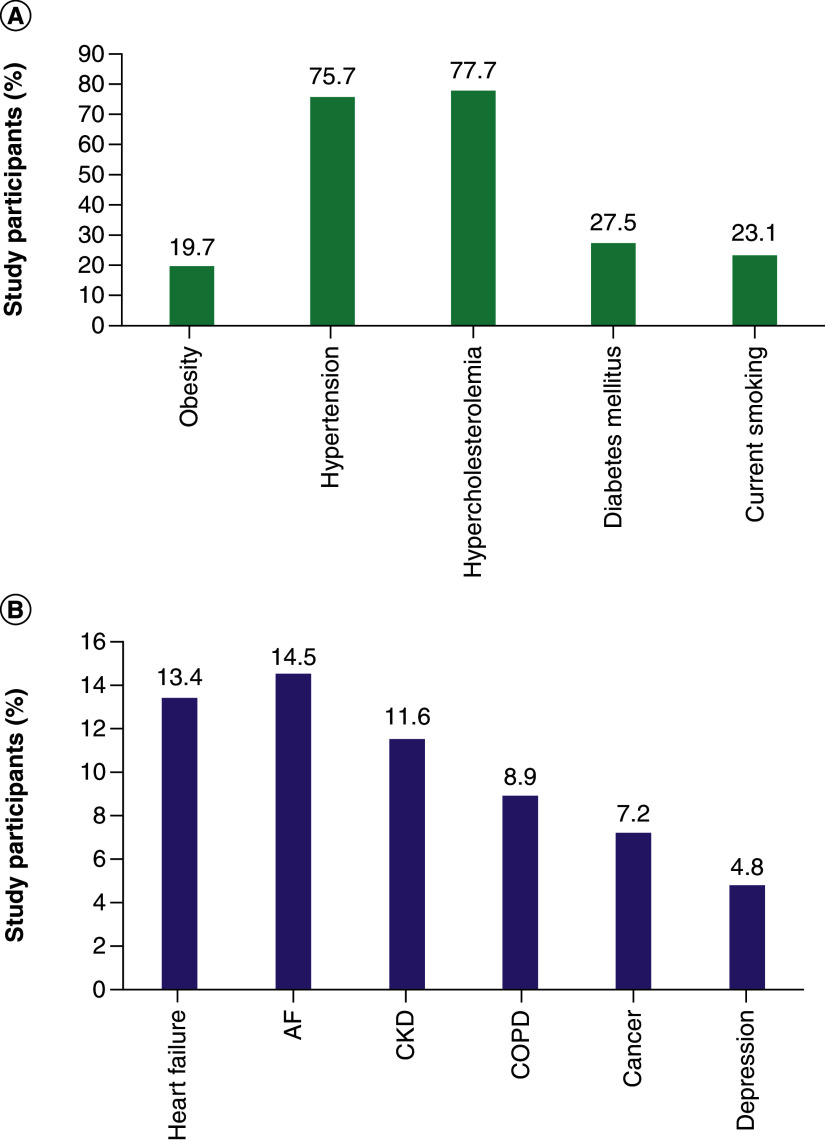
Multimorbidity in 4790 patients with atherothrombotic disease in the BRING-UP Prevention study in Italy. The prevalence of **(A)** cardiovascular risk factors and **(B)** comorbidities [54]. AF: Atrial fibrillation; CKD: Chronic kidney disease; COPD: Chronic obstructive pulmonary disease.

Data from the ongoing, observational BRING-UP Prevention study being conducted at 189 cardiology centers across Italy illustrates the multimorbidity present in the CVaD patient population [54]. Patients are included in the study if they have stable atherothrombotic disease, which includes CAD (history of ACS or revascularization), cerebrovascular disease (history of ischemic stroke or carotid revascularization) or PAD (intermittent claudication with objective evidence of PAD, peripheral revascularization or lower leg amputation as a result of PAD). Figure 4A shows the extent to which these patients have multiple cardiovascular risk factors and Figure 4B the extent of vascular disease and other comorbidities. Among the 4790 patients enrolled during the first phase of this study, 4694 (98.0%) had CAD, 292 (6.1%) had cerebrovascular disease and 329 (6.9%) had PAD, with some patients having involvement in more than one vascular bed [54]. About half of the patients in the BRING-UP Prevention cohort are overweight, with BMI >27 kg/m^2^ in 42.7% (n = 2044), and one in five patients is obese (BMI ≥30 kg/m^2^, n = 945 [19.7%]). Although the mean systolic BP (SBP) for the overall cohort was within the target range (120.9 mmHg), 2298 patients (48.0%) had SBP ≥130 mmHg [54]. Around a third of patients (32.6%) had LDL cholesterol levels below the level recommended by the European Society of Cardiology (<55 mg/dl), and 47.2% of patients had LDL cholesterol levels in excess of 70 mg/dl [54]. These data demonstrate that considerable proportions of patients with multimorbidity do not meet therapeutic targets for risk factor control.

Similar findings have been made in the international SNAPSHOT observational studies in patients with hypertension, which appear to confirm that many patients with one CVaD risk factor have multiple comorbidities (Table 3) [55–57]. Further, the majority of these patients (65–87%) are at very high risk of a CVaD event [55–57]. SNAPSHOT investigators suggested that physicians consistently underestimate patients' risk levels [55,57]. Although the data from the SNAPSHOT studies have not yet been fully published, initial indications suggest that most patients do not have BP, LDL cholesterol or glycemic indices controlled to guideline-recommended levels [55,57].

**Table 3.** Preliminary data from the SNAPSHOT observational studies among patients with hypertension in different countries.

	Europe [55]	South America [56,57]
Countries	Bulgaria, Croatia, Georgia, Romania, Serbia, Spain	Colombia
n	9443	459
Males, %	43	42
Type 2 diabetes, %	32.8	100.0^†^
Dyslipidemia, %	80.8	85.6
Overweight/obesity, %	81.6	73.4
Current/former smoking	29.5	23.8
≥2 risk factors^‡^, %	Not reported	44.4
Very high risk^§^, %	86.8	65.4

† The Colombian SNAPSHOT study was undertaken in patients with hypertension and Type 2 diabetes.

‡ Risk factors defined as male sex, age ≥65 years, obesity, and smoking.

§ Using the SCORE2 [58] and SCORE2-OP [59] risk calculator.

### Missed opportunities

The studies discussed above highlight consistently missed therapeutic opportunities in the management of patients with CVaD and/or CVeD. In our view there are two key reasons: therapeutic inertia and patient treatment adherence.

#### Therapeutic inertia

The American Heart Association published a Call to Action in 2019, identifying points in the therapeutic continuum where opportunities to improve outcomes are missed, including failure to diagnose and modify risk factors such as hypertension and dyslipidemia, failure to elicit and monitor patient’s goals and needs, failure to use evidence-based interventions, and eventually failure to provide advanced, supportive and palliative care in the final stages of CVaD [8]. One reason for this (as demonstrated in the SNAPSHOT data) is that physicians often underestimate a patient’s CV risk and overestimate the extent to which risk factors such as BP and lipid levels are controlled [60,61]. This underestimation of risk and overestimation of control may lead to therapeutic inertia, whereby treatment is not appropriately escalated to achieve target levels.

The contribution of therapeutic inertia to poor patient outcomes is being addressed internationally by a number of groups [62,63]. Drawing on the available research, these initiatives highlight a number of key determinants of improving therapeutic inertia, including collaborative and team-based care with shared decision-making and personalized management plans [62–64], and increased use of guideline-recommended therapies [62–64].

**Figure 5.**
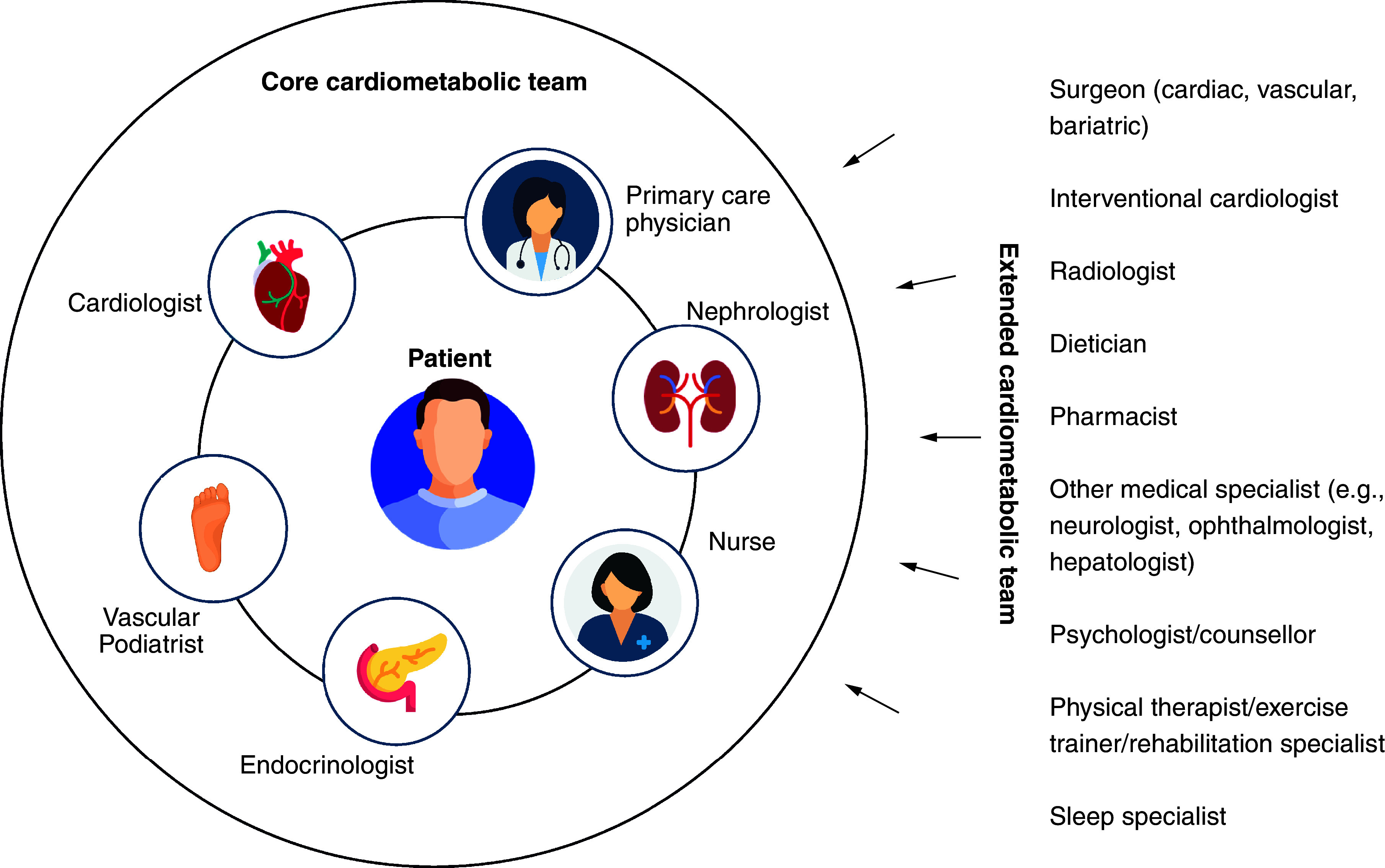
Example of the structure and members of a multidisciplinary team to manage patients with cardiometabolic disease.

Collaborative care requires the formation of alliances between individual HCPs; in a process the American Diabetes Association has termed “collaborative barrier busting” [63]. The key is to engage a core multidisciplinary team around the patient and co-opt additional HCPs into the patient’s care when the need arises or to address specific comorbidities [65]. An example of what these teams may look like is shown in Figure 5; ideally the core care team will include a cardiologist, vascular podiatrist, endocrinologist, nurse(s), nephrologist as well as their primary care physician, with multiple other specialists involved in the extended cardiometabolic team. In order to do this, systems have to be developed that encourage constructive communication with the patient and between HCPs, which may include the use of information technology to share information [65]. In addition, medical education at both the undergraduate and postgraduate level should be revised to include a more holistic and multidisciplinary approach to cardiometabolic disease prevention and management [6].

Such systemic changes often take time to implement, but in the meantime, steps can be taken to reduce therapeutic inertia by following international guidelines for early use of combination therapy. European Society of Hypertension (ESH) and European Society of Cardiology (ESC) guidelines now both recommend starting most patients with newly diagnosed hypertension on dual combination therapy with an angiotensin-converting enzyme inhibitor or angiotensin receptor blocker, calcium channel blocker or diuretic [66,67]. If this combination is ineffective, the guidelines recommend adding a third agent [66,67]. In patients with overt chronic CAD (angina pectoris), clinical guidelines also recommend the use of combination therapy, tailored to the patient’s clinical profile, hemodynamic status and the presence of vasospasm or microvascular dysfunction [68]. Recommended add-on therapy for chronic CAD includes trimetazidine [68], which (when added to metoprolol) has been shown to significantly reduce the incidence of anginal attacks and improve exercise tolerance in patients with angina, compared with metoprolol monotherapy [69]. The combination of trimetazidine + propranolol was also more effective than isosorbide dinitrate + propranolol for reducing anginal attacks [70].

#### Adherence

Adherence is difficult to measure but is commonly based on measures of prescription refills, with 80% adherence being the threshold defined as having a clinically relevant effect on outcomes in chronic disease [71]. Using this threshold, data indicate that adherence to cardiovascular preventive therapies is suboptimal, with between 40 and 70% of patients taking their medication as prescribed >80% of the time [50,72–75]. Moreover, about 50% of patients discontinue such therapies within the first 12 months [74]. The low adherence rates among patients with cardiovascular risk factors or CVaD led to the first World Adherence Day on 27 March 2025, drawing attention to the need for patients to take their medication as prescribed to optimize outcomes.

There are multiple factors associated with adherence related to the patient’s circumstances and health, the patient–doctor relationship, the healthcare system and the treatment prescribed (Table 4) [76]. It is important for HCPs to question their patients about adherence and identify potential barriers to adherence that can be addressed. One potential barrier is pill burden.

**Table 4.** Causes of medication nonadherence.

Healthcare factors	Treatment-related factors	Patient-related factors	Condition-related factors
• Poor patient–physician relationship/communication • Fragmented care • Lack of follow-up • Short consultation times • Healthcare professional knowledge gap about adherence (generally and specific to the individual patient)	• Complex regimens/pill burden • Need for titration/dose adjustment • Adverse events (actual or perceived) • Lack of feedback • Stigma (e.g., for injectable medications) • Interference with daily life (e.g., medication timing in relation to food and frequent urination caused by diuretics)	• Age • Cognitive impairment/forgetfulness • Physical impairments (e.g., hearing, visual and manual dexterity) • Patient expectations and goals • Psychosocial factors (e.g., stress, depression and anxiety) • Language barriers • Social embarrassment/stigma • Socioeconomic circumstances (e.g., cost of medication and insurance coverage) • Health literacy/educational level • Access to pharmacy • Social support (e.g., spouse, family and friends)	• Chronicity • Lack of symptoms (e.g., for lipid levels and blood pressure) • Long asymptomatic phases (e.g., if condition associated with flares)
Data are from Nelson *et al.* [76].			

Adherence tends to be lower in patients taking more than one type of cardiovascular therapy, i.e., patients taking antihypertensive and lipid-lowering therapy have better adherence for one type of treatment than for both [75,77]. Pill burden may contribute to this phenomenon, since adherence has been shown to decrease as the number of prescription medicines increase [72]. One easy approach to enhancing adherence is to simplify the treatment regimen, by discontinuing unnecessary medications, prescribing agents that are active against more than one cardiovascular risk factor (e.g., treatments for diabetes that cause weight loss, treatments for hypertension that also have renoprotective effects) [23], prescribing once daily medications wherever possible, and considering single-pill combination (SPC) therapies to reduce the pill burden [76].

SPCs have been shown to improve adherence and outcomes across a range of cardiometabolic indications, including hypertension [78], dyslipidemia [79,80] and diabetes [81,82] (Table 5). The use of SPCs is now a class I recommendation in European guidelines for hypertension [66] and chronic CAD [68], to enhance adherence and persistence.

**Table 5.** Comparison of adherence to single-pill combination therapy versus free combination therapy^§^.

Cardiometabolic indication	Study	Treatment	Comparator groups (n)	% patients adherent^¶^	Patient outcomes	Ref.
Hypertension	Healthcare database study of matched cohorts (Italy)	PER/IND/AML	SPC (n = 12,150) vs FCT (n = 6105)	59.9 vs 26.9%^‡^	Mortality (1000-person/year): 29.9 vs 33.7^†^ Composite death/CV event (1000-person/year): 105.8 vs 139.0^‡^	[78]
Dyslipidemia	Healthcare database study (Italy)	ROS/EZE	SPC (n = 25,886) vs FCT (n = 7309)	*Overall* 56.8% vs 44.5%^‡^ *By CV risk level* Very high: 65.4 vs 50.4%^‡^ High: 54.7 vs 42.7%^‡^ Other: 43.5 vs 35.9%^‡^	% pts achieving LDL-C targets^#^ by CV risk level: Very high: 35.4 vs 23.8%^‡^ High: 46.9 vs 23.1%^‡^ Other: 71.6 vs 49.5%^‡^	[80]
	Healthcare database study (Italy)	ROS/EZE	SPC (index period) vs FCT (pre-index period) (n = 1219)	75.2 vs 51.8%^‡^	–	[79]
Diabetes	National registry matched cohort study (Sweden)	Metformin + (SGLT2i/DPP4i/TZD)	SPC (n = 13,883) vs FCT (n = 13,883)	68.6 vs 46.5%	HF rate (per 1000-person years): 8.1 vs 9.2 (HR: 0.88; 95% CI: 0.79–0.99)	[82]

† p < 0.05.

‡ p < 0.001.

§ Referred to also as loose-dose combination therapy.

¶ Patients were considered adherent at the following thresholds: PDC ≥80% [78]; PDC >80% [82], and PDC >75% [79,80].

# LDL-C targets were <55 mg/dL in very high CV risk group, <70 mg/dL in the high-risk group and <116.mg/dL in other risk group [80].

Data are from selected representative studies.

AML: Amlodipine; CI: Confidence interval; CV: Cardiovascular; DPP4i: Dipeptidyl peptidase 4 inhibitor; EZE: Ezetimibe; FCT: Free combination therapy; HF: Heart failure; HR: Hazard ratio; IND: Indapamide; LDL-C: Low-density lipoprotein cholesterol; PDC: Proportion of days covered; PER: Perindopril; ROS: Rosuvastatin; SGLT2i: Sodium-glucose cotransporter 2 inhibitor; SPC: Single-pill combination; TZD: Thiazolidinedione.

Other approaches to enhancing adherence include digital tools, such as smart phone apps, wearable devices and text messaging [66,68,76]. These digital tools have proven to significantly improve outcomes in patients with chronic cardiovascular conditions, particularly hypertension [83–85], with data showing that adherence is maximized by initiatives that include more than one type of e-health intervention [72]. As well as providing patients with reminders and feedback, these digital tools can enhance health literacy [86] and help to overcome some of the barriers to risk factor management seen in underserved communities [87].

## Conclusions

Addressing the escalating global burden of CVaD necessitates a paradigm shift toward a more integrated, holistic approach to patient management. Recognizing the interconnectedness of arterial and venous vascular conditions, along with shared risk factors such as diabetes, obesity, hypertension and socioeconomic determinants, is crucial for early identification and comprehensive treatment. Clinicians must prioritize timely diagnosis, aggressive risk factor modification and adherence to evidence-based therapies, leveraging multidisciplinary teams and innovative strategies like single-pill combinations and digital health tools to combat therapeutic inertia and improve adherence. Future research should focus on elucidating the role of CVeD in cardiovascular risk, developing targeted interventions, and implementing systemic healthcare reforms that promote proactive, personalized care. Ultimately, such efforts will optimize outcomes, reduce disability and mitigate the devastating impact of CVaD worldwide.

## Summary points

- Cardiovascular disease (CVaD) is a prevalent health condition frequently accompanied by chronic venous disease (CVeD).
- CVaD risk factors such as obesity, diabetes and hypertension adversely affect the structure and function of arteries and veins.
- Damage to the veins, known as CVeD, shares common risk factors and pathogenic pathways with CVaD.
- Mainstays of treatment for CVeD are exercise, compression therapy, venoactive drugs and surgery.
- Unfortunately, patients with CVaD and/or CVeD or their risk factors are frequently underdiagnosed and untreated.
- Patients may not achieve guideline-recommended targets for disease control of hypertension, dyslipidemia and diabetes due to therapeutic inertia and/or poor medication adherence.
- A coherent approach is needed to identify and manage shared risk factors and comorbidities.
- The authors propose that multidisciplinary teams formulate a clear strategy for patient care, that they utilize treatments for which there is strong evidence of effectiveness, and that tools to maximize adherence be used, particularly in patients with multiple risk factors or comorbidities.
